# Pre-eclampsia in pregnancy and subsequent risk for breast cancer

**DOI:** 10.1038/sj.bjc.6600581

**Published:** 2002-10-21

**Authors:** L J Vatten, P R Romundstad, D Trichopoulos, R Skjærven

**Affiliations:** Department of Community Medicine and General Practice, Norwegian University of Science and Technology, Trondheim, Norway; Department of Epidemiology, Harvard School of Public Health, Boston, Massachusetts, USA; The Medical Birth Registry and Section for Medical Statistics, University of Bergen, Norway

**Keywords:** pre-eclampsia, hypertension in pregnancy, breast cancer

## Abstract

Women who experience pre-eclampsia or hypertension during pregnancy may have a reduced risk for breast cancer later in life. The evidence is based on case–control studies, and here we report the results of a cohort study exploring the link between pre-eclampsia and gestational hypertension diagnosed in the first pregnancy and subsequent risk for breast cancer. We combined information from the Medical Birth Registry and the Cancer Registry in Norway, which are both nation-wide. Between 1967, when the birth registry was established, and 1998, 694 657 women were recorded with a first birth, and classified according to whether pre-eclampsia and/or hypertension was diagnosed in the first pregnancy. Linkage to the Norwegian Cancer Registry identified 5474 new cases of breast cancer diagnosed subsequently to their first delivery. Compared to other parous women, women with pre-eclampsia and/or hypertension diagnosed in their first pregnancy had 19% lower risk (95% confidence interval, 9 to 29%) for breast cancer, after adjustment for attained age, calendar period of diagnosis, age at first birth, and parity. This result was similar for term and preterm deliveries, across the range of offspring birth weight, and for pre- and postmenopausal women. These results suggest that the pathophysiology surrounding pre-eclampsia and gestational hypertension plays an important role in breast cancer etiology. A better understanding of the underlying processes could provide an insight into the pathogenesis of breast cancer.

*British Journal of Cancer* (2002) **87**, 971–973. doi:10.1038/sj.bjc.6600581 www.bjcancer.com

© 2002 Cancer Research UK

## 

Several risk factors for breast cancer have been identified (Kelsey, 1993), but few of them are linked to identifiable mechanisms that allow a clear insight into the natural history of breast cancer. There have been studies indicating that pre-eclampsia or hypertension in pregnancy is associated with subsequent reduction in breast cancer risk in the mother (Polednak and Janerich, 1983; Troisi *et al*, 1998; Thompson *et al*, 1989; Cohn *et al*, 2001). If confirmed, this association could be particularly useful in understanding the pathogenesis of breast cancer (Hoover and Troisi, 2001), because pre-eclampsia has an identifiable, if not yet fully identified, pathophysiology (Roberts and Cooper, 2001). The earlier investigations of the association of pre-eclampsia or pregnancy hypertension with breast cancer risk (Polednak and Janerich, 1983; Thompson *et al*, 1989; Troisi *et al*, 1998; Cohn *et al*, 2001), however, have all had a case–control design that is not immune to subtle biases. We report here the results of a cohort study exploring the link between pre-eclampsia and/or hypertension in pregnancy and subsequent breast cancer risk in the mother.

## MATERIALS AND METHODS

Data were derived from the Norwegian Medical Birth Registry that comprises all births since 1967, and the Norwegian Cancer Registry, which has registered incident cancers since 1953. Midwives and doctors have to fill in a standardised form to notify the birth registry about each birth that takes place in the country, and the reporting of cancer by doctors or hospital departments to the cancer registry is also mandatory. The unique identification number of Norwegian citizens enabled linkage between the birth registry and the cancer registry for cancer follow-up.

We have identified cases of pre-eclampsia and/or hypertension induced by pregnancy as indicated in the standardised form, and the women were accordingly categorised as having been diagnosed with at least one of these conditions (Lie *et al*, 1998). In the analyses, we further subdivided the women according to whether they had a premature delivery (<37 weeks' gestation) or not, according to offspring's birth weight (in three categories: small, appropriate, or large for gestation), and according to age at diagnosis (cut-off at 50 years to approximate pre- and postmenopausal status).

We used each woman's identification number to link the women registered at the medical birth registry to the national cancer registry in order to identify women who had developed cancer subsequent to giving birth. In total, 756 414 women have been recorded with a first birth in the birth registry between 1967 and 1998. Of these, 1556 women were excluded from analysis, either because they had a diagnosis of cancer recorded prior to their first birth, or because they had emigrated and could not be traced during follow-up. We also excluded 60 201 women who had incomplete information on offspring birth weight or length of gestation at birth. Thus, we have followed 694 657 women from their first birth until the diagnosis of cancer, until death from any cause, or to the end of follow-up (December 31, 1998), whichever occurred first.

We compared breast cancer risk in women who had experienced pre-eclampsia and/or hypertension induced by their first pregnancy with the risk in other parous women, and adjusted for attained age (nine categories), calendar period of diagnosis (three categories), age at first birth (five categories), and total number of births (five categories). In the multivariate analyses, we applied Poisson regression modelling, using the Epicure software (Epicure, Seattle, WA, USA: Hirosoft Int Corp, 1993), and we used SPSS, version 10.05 (SPSS, Inc., Chicago, IL, USA), for the descriptive analyses.

## RESULTS

During follow-up, 5474 cases of breast cancer were diagnosed among the 694 657 parous women. Of these cases, 280 occurred among women who were diagnosed with pre-eclampsia and/or hypertension in their first pregnancy. These women had a 19% lower risk of subsequent breast cancer than the remaining women (rate ratio 0.81, 95% confidence interval 0.71 to 0.91), after adjustment for attained age, calendar period of diagnosis, age at first birth, and parity (Table 1

**Table 1:**
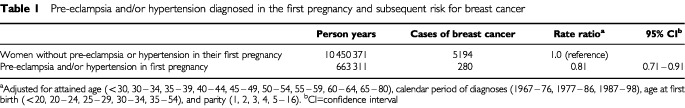
Pre-eclampsia and/or hypertension diagnosed in the first pregnancy and subsequent risk for breast cancer

).

We explored whether the association between pre-eclampsia and breast cancer risk could be modified by length of gestation (preterm or term delivery), offspring birth weight, or the woman's age (cut-off at 50 years). The results show that the pre-eclampsia-related reduction of breast cancer risk did not substantially differ across these subgroups (Table 2

**Table 2:**
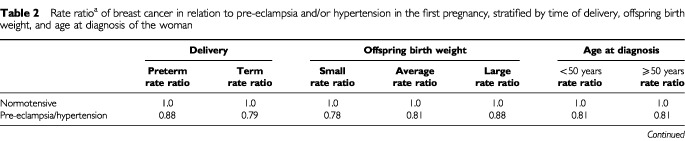
Rate ratio^a^ of breast cancer in relation to pre-eclampsia and/or hypertension in the first pregnancy, stratified by time of delivery, offspring birth weight, and age at diagnosis of the woman

).

## DISCUSSION

We found that women who were diagnosed with pre-eclampsia and/or hypertension in their first pregnancy were at reduced risk of breast cancer, compared to other women who did not develop pre-eclampsia or hypertension during their first pregnancy. The inverse association has also been found in the previously reported case–control studies (Polednak and Janerich, 1983; Troisi *et al*, 1998; Thompson *et al*, 1989; Cohn *et al*, 2001).

The prospective nature of this study all but excludes selection or information bias as plausible explanations for the findings, and the large number of women makes it unlikely that chance can play an important role.

The clinical manifestations of pre-eclampsia are heterogeneous, and this could indicate that the condition comprises different biological entities (Ness and Roberts, 1996). If so, one might expect that the association between pre-eclampsia and breast cancer risk would differ among subtypes of pre-eclampsia. However, by exploring the association between different characteristics of pre-eclampsia and breast cancer risk, we found that the reduced risk for breast cancer was consistent between term and preterm deliveries, across the range of the offspring's birth weight, and between pre- and postmenopausal women.

Our analysis focused on the first pregnancy, with adjustment for total ascertainable parity. Although less common, some women will have developed pre-eclampsia or hypertension also during higher order pregnancies, and because of this, the pre-eclampsia-related protection against breast cancer in our study could have been somewhat underestimated.

A central characteristic of pre-eclampsia is an abnormal implantation that has been attributed to a shallow connection between the placenta and the endometrium (Roberts and Cooper, 2001). A combination of genetic susceptibility and abnormal placentation is thought to constitute the basis for pre-eclampsia, and clinically, the syndrome is characterised by hypertension and proteinuria. In women who develop pre-eclampsia, there may be a disturbance in the fundamental balance between steroid and other pregnancy hormones, with relatively lower oestrogen, and higher androgen levels (Innes and Byers, 1999). Since pre-eclampsia conveys some protection against breast cancer risk, a better understanding of its pathophysiology could also provide an insight into the pathogenesis of breast cancer.
